# A young man diagnosed with chronic thromboembolic pulmonary hypertension after COVID-19 infection: A case report

**DOI:** 10.1016/j.jccase.2025.01.001

**Published:** 2025-01-31

**Authors:** Mitsumasa Akao, Kayoko Kubota, Sunao Miyanaga, Kokoro Mitsuyoshi, Mitsuru Ohishi

**Affiliations:** Department of Cardiovascular Medicine and Hypertension, Graduate School of Medical and Dental Sciences, Kagoshima University, Kagoshima, Japan

**Keywords:** COVID-19, Chronic thromboembolic pulmonary hypertension, Shortness of breath

## Abstract

Chronic thromboembolic pulmonary hypertension (CTEPH) is a rare disease characterized by pulmonary hypertension (PH), resulting from stenosis or occlusion of the pulmonary arteries owing to an organic thrombus that obstructs blood flow in the pulmonary arteries. The prognosis for untreated patients is poor; however, it has improved significantly with the advent of treatments such as PH-targeted medical therapy and pulmonary balloon angioplasty, in addition to pulmonary endarterectomy. Nevertheless, the exact mechanisms underlying this disease remain unknown. Recently, a close association between coronavirus disease 2019 (COVID-19) and thrombosis has been detected, with the risk of venous thrombosis complications increasing after COVID-19 infection; however, few studies have reported on the association between COVID-19 and CTEPH. Herein, we present the case of a young man who developed CTEPH after a mild COVID-19 infection, despite the lack of an obvious thrombophilic predisposition. We conclude that if a patient develops chronic shortness of breath symptoms after a COVID-19 infection, it is important to investigate not only the COVID-19 sequelae, but also the presence of other diseases such as pulmonary artery thrombosis.

**Learning objective:**

Coronavirus disease 2019 (COVID-19) infection frequently causes abnormal blood coagulation and is closely related to thrombosis. Although pulmonary embolism is a frequent complication of venous thrombosis, few studies have reported an association between COVID-19 and chronic thromboembolic pulmonary hypertension (CTEPH). Our patient developed CTEPH after COVID-19 infection. It is important to examine organic abnormalities, before diagnosing persistent dyspnea as a COVID-19 sequela.

## Introduction

Chronic thromboembolic pulmonary hypertension (CTEPH) is a rare disease that causes pulmonary hypertension (PH) because of stenosis and occlusion of the pulmonary artery by an organic thrombus [1]. According to the current definition, CTEPH is characterized by a mean pulmonary artery pressure (mPAP) value >20 mmHg, pulmonary artery wedge pressure (PAWP) value ≤15 mmHg, and pulmonary vascular resistance (PVR) value >2 Wood units (WU). In addition, at least 3 months of anticoagulation therapy is required to make a diagnosis [2]. The symptoms of CTEPH include shortness of breath, edema, fatigue, and syncope, with untreated patients exhibiting poor prognosis. This disease has a low prevalence; in Japan, it is more prevalent among women than men, with a predilection for individuals in their 60s and 70s. The prognosis has improved substantially since the introduction of PH-targeted therapy and pulmonary balloon angioplasty, in addition to pulmonary endarterectomy. However, the exact mechanisms underlying this disease remain unknown.

Recently, coronavirus disease 2019 (COVID-19) infection has been reported to increase the risk of venous thrombosis [3], indicating a close association between COVID-19 and thrombosis. However, only a few studies have reported on the association between CTEPH and COVID-19. Herein, we report the case of a young man who developed CTEPH after a COVID-19 infection.

## Case report

**Fig. 1:**
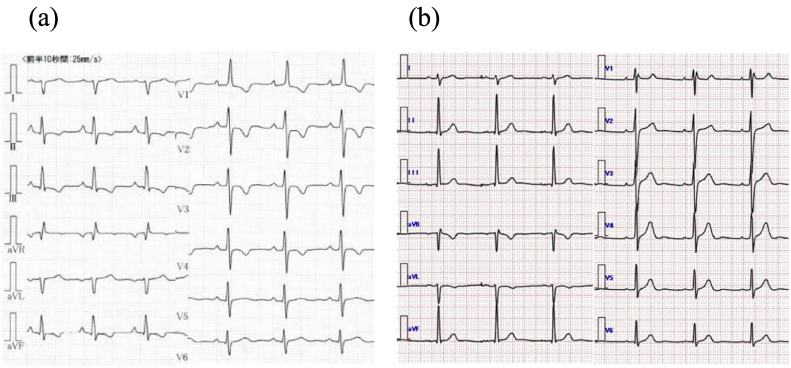
Electrocardiography at (a) symptom onset and (b) after completion of pulmonary balloon angioplasty. The electrocardiography results show improvement of R wave enhancement in V1 and negative T waves in II, III, aVF, and V1–V4.

**Fig. 2:**
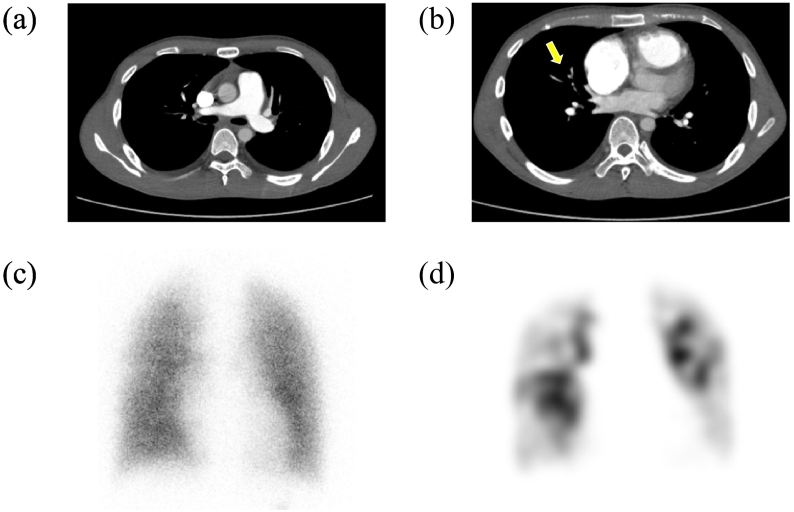
Contrast computed tomography and pulmonary scintigraphy images. Panels (a) and (b) show contrast-enhanced computed tomography at the onset of symptoms. These images show no thrombus in the main pulmonary artery trunk; however, an area of poor contrast is present in the pulmonary artery of the right middle lobe (yellow arrow). Panel (c) shows pulmonary ventilation scintigraphy and panel (d) shows pulmonary perfusion scintigraphy at the time of diagnosis. Multiple mismatch findings are observed between the two images.

**Fig. 3:**
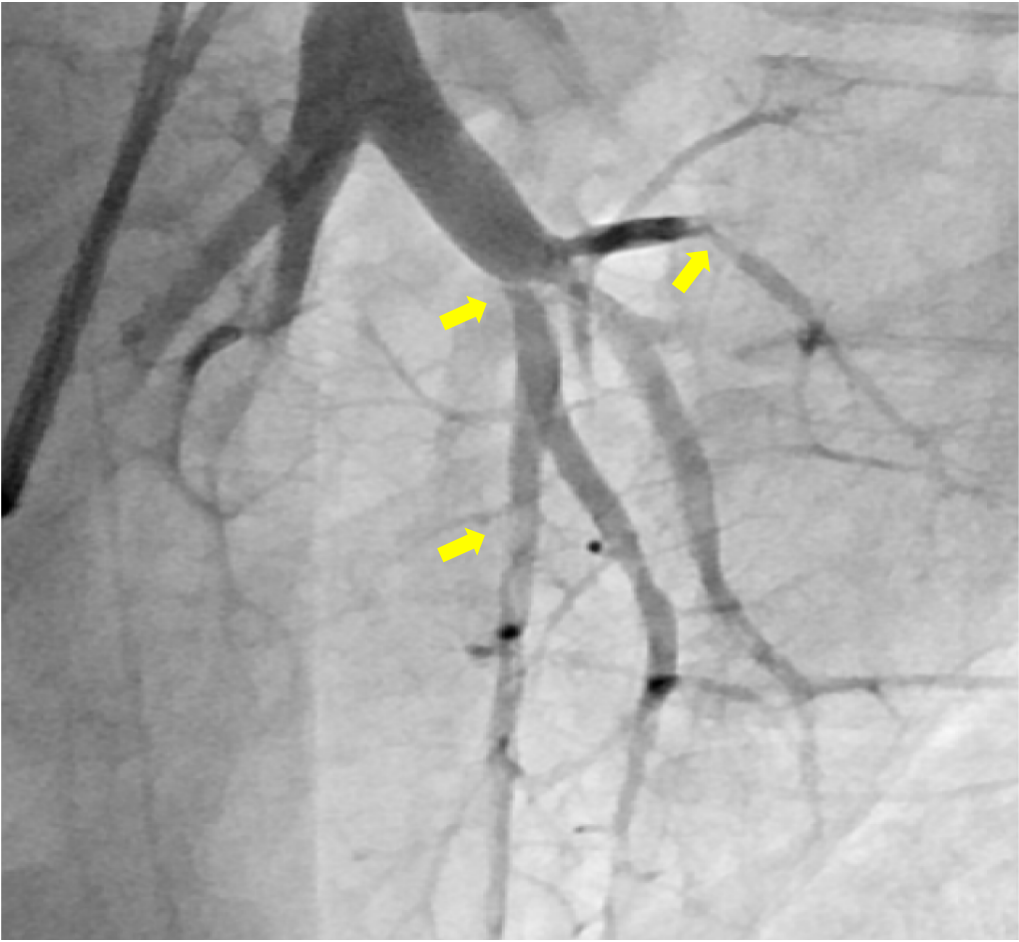
Pulmonary arteriography at the time of diagnosis. This image shows web and silt lesions detected in the pulmonary artery (yellow arrows).

In this case, the patient was a healthy 19-year-old male. No radiographic or electrocardiographic abnormalities were observed in the patient's high school medical examination reports. The patient had no history of COVID-19 vaccination and contracted COVID-19 in May 2021; however, the disease was mild. At that time, a plain computed tomography (CT) scan showed no enlargement of the pulmonary artery or right ventricle. However, 6 months after discharge from the hospital, the patient experienced gradually worsening shortness of breath; by 2022, the patient had begun to faint repeatedly. The patient also developed edema of the lower legs and decided to visit our hospital in January 2022. Blood test results showed elevated D-dimer (3.09 μg/mL), and echocardiography radiographs showed right ventricular load dysfunction. Electrocardiography findings showed right-axis deviation and right ventricular hypertrophy (Fig. 1). Although contrast-enhanced CT showed no thrombus in the central region (Fig. 2), lung perfusion scintigraphy revealed multiple defects in both lungs, leading to a diagnosis of pulmonary artery thromboembolism. Therefore, anticoagulation therapy was initiated. Three months later, the patient visited our hospital for follow-up and still had symptoms of World Health Organization Functional Class II dyspnea. However, no other symptoms associated with PH were observed. We performed a variety of diagnostic tests, and the blood test results revealed elevated levels of B-type natriuretic peptide (103.4 pg/mL) and no evidence of protein C or S deficiency or antiphospholipid syndrome. Chest radiography showed enlarged pulmonary arteries; therefore, contrast-enhanced CT and lower extremity venous ultrasound were performed, but no deep vein thrombosis or other thrombosis was observed. Respiratory function tests and arterial blood gas analysis showed no obvious abnormalities; however, the 6-min-walk test distance was short (335 m). Echocardiography showed right ventricular enlargement and an elevated right ventricular systolic pressure of 60.4 mmHg. Moreover, right heart catheterization revealed a PAWP value of 7 mmHg, mPAP value of 38 mmHg, cardiac index of 2.1 L/min/m^2^, and PVR value of 8.7 WU. Pulmonary ventilation and perfusion scintigraphy revealed mismatched findings (Fig. 2), and pulmonary angiography revealed multiple web and slit lesions in the bilateral pulmonary arteries (Fig. 3). Screening for PH ruled out other causes, and we arrived at the diagnosis of CTEPH. Subsequently, we performed a total of five percutaneous pulmonary artery angioplasties after initiating drug therapy with riociguat. As a result, the mPAP improved to 22 mmHg, PVR decreased to 2.2 WU, 6-min-walk distance increased to 457 m, electrocardiography abnormalities improved, and shortness of breath disappeared.

## Discussion

This report describes the case of a young man who was diagnosed with CTEPH >6 months after infection with COVID-19. In Japan, CTEPH is diagnosed more frequently in women than in men, with a common age of onset of 60–70 years [4]; therefore, CTEPH diagnosis in young men, such as the patient described herein, is relatively uncommon. Although a previous study has reported on CTEPH in young patients, these patients were also diagnosed with antiphospholipid antibody syndrome [5]. In contrast, the patient in the present study had a history of COVID-19 infection.

In previous reports, COVID-19 infection was shown to be associated with increased thrombosis. For instance, a meta-analysis of 42 studies with a total of 8271 patients reported that the frequencies of venous thromboembolism and pulmonary embolism in patients with a history of COVID-19 infection were approximately 21 % and 13 %, respectively [3]. The present case occurred during the delta-variant epidemic; notably, Manzur-Pineda et al. reported that the delta variant has a 1.36-times higher risk of thrombotic complications than the non-delta variants [6]. In their study [6], the time from admission to confirmation of a thrombotic event was very short, with a median of 4 days (range, 1–12 days); however, in the present case, the patient became aware of symptoms approximately 6 months after COVID-19 infection, and a diagnosis was made almost 1 year after the infection. In a previous study, CTEPH developed in 3.8 % of patients after severe pneumonia due to COVID-19 [7]; however, in our patient, pneumonia was mild, and oxygen administration was not required.

Although the mechanism of CTEPH development after COVID-19 is unclear, previous studies have reported that neutrophil extracellular traps (NETs) have an important role in COVID-19-associated thrombosis. NETs are web-like structures composed of deoxyribonucleic acid and proteins that are shed from neutrophils; these structures serve as an immune defense mechanism that functions to capture pathogens. Previous studies have suggested that NETs may contribute to damage in vascular endothelial cells and activate blood coagulation pathways in the microvasculature [8]. Several studies have reported increased neutrophil count and NET formation in patients with CTEPH. One such study showed that activated fibroblasts differentiated from NET-induced monocytes exhibit cellular phenotype similar to those of fibroblasts observed in the occluded vessels of patients with CTEPH [9]. Therefore, NETs may be involved in the association between COVID-19 and CTEPH.

When COVID-19 leads to sequelae, these symptoms may persist for a long time, which is not uncommon. Miyazato et al. reported that 17.5 % of patients with COVID-19 had shortness of breath 60 days after symptom onset [10]. However, it is difficult to distinguish sequelae or organic abnormalities from clinical symptoms alone, and it is important to identify the cause, considering the possibility of pulmonary embolism. Without a thorough examination, a case of persistent shortness of breath symptoms in a patient with non-severe COVID-19 infection could be considered to only be a sequela of COVID-19, and a thrombotic condition could be missed.

Thus, the present case report highlights the importance of considering CTEPH and other diseases in patients with a history of COVID-19 infection, even among young patients.

## Patient permission/consent statement

The patient provided informed consent for publication of this report and all accompanying data.

## Declaration of competing interest

The authors declare that there is no conflict of interest.
